# Prescribing of direct oral anticoagulants and warfarin to older people with atrial fibrillation in UK general practice: a cohort study

**DOI:** 10.1186/s12916-021-02067-5

**Published:** 2021-08-31

**Authors:** Anneka Mitchell, Julia Snowball, Tomas J. Welsh, Margaret C. Watson, Anita McGrogan

**Affiliations:** 1 Department of Pharmacy and Pharmacology, University of Bath, Bath, BA2 7AY UK; 2 Pharmacy Research Centre, University Hospital Southampton, Southampton, SO16 6YD UK; 3 Research Institute for the Care of Older People (RICE), Bath, BA1 3NG UK; 4 Institute of Clinical Neurosciences, University of Bristol, Bristol, BS8 1TH UK; 5 Strathclyde Institute of Pharmacy and Biomedical Sciences, University of Strathclyde, Glasgow, G4 0RE UK

**Keywords:** Older people, Anticoagulant, Atrial fibrillation, Warfarin, Dabigatran, Rivaroxaban, Apixaban, Edoxaban

## Abstract

**Background:**

Anticoagulation for stroke prevention in atrial fibrillation (AF) has, historically, been under-used in older people. The aim of this study was to investigate prescribing of oral anticoagulants (OACs) for people aged ≥ 75 years in the UK before and after direct oral anticoagulants (DOACs) became available.

**Methods:**

A cohort of patients aged ≥ 75 years with a diagnosis of AF was derived from the Clinical Practice Research Datalink (CPRD) between January 1, 2003, and December 27, 2017. Patients were grouped as no OAC, incident OAC (OAC newly prescribed) or prevalent OAC (entered study on OAC). Incidence and point prevalence of OAC prescribing were calculated yearly. The risk of being prescribed an OAC if a co-morbidity was present was calculated; the risk difference (RD) was reported. Kaplan-Meier curves were used to explore persistence with anticoagulation. A Cox regression was used to model persistence with warfarin and DOACs over time.

**Results:**

The cohort comprised 165,596 patients (66,859 no OAC; 47,916 incident OAC; 50,821 prevalent OAC). Incidence of OAC prescribing increased from 111 per 1000 person-years in 2003 to 587 per 1000 person-years in 2017. Older patients (≥ 90 years) were 40% less likely to receive an OAC (RD −0.40, 95% CI −0.41 to −0.39) than younger individuals (75–84 years). The likelihood of being prescribed an OAC was lower with a history of dementia (RD −0.34, 95% CI −0.35 to −0.33), falls (RD −0.17, 95% CI −0.18 to −0.16), major bleeds (RD −0.17, 95% CI −0.19 to −0.15) and fractures (RD −0.13, 95% CI −0.14 to −0.12). Persistence with warfarin was higher than DOACs in the first year (0–1 year: HR 1.25, 95% CI 1.17–1.33), but this trend reversed by the third year of therapy (HR 0.75, 95% CI 0.63–0.89).

**Conclusions:**

OAC prescribing for older people with AF has increased; however, substantial disparities persist with age and co-morbidities. Whilst OACs should not be withheld solely due to the risk of falls, these results do not reflect this national guidance. Furthermore, the under-prescribing of OACs for patients with dementia or advancing age may be due to decisions around risk-benefit management.

**Trial registration:**

EUPAS29923﻿. First registered on: 27/06/2019.

**Supplementary Information:**

The online version contains supplementary material available at 10.1186/s12916-021-02067-5.

## Background

Atrial fibrillation (AF) is a common cardiac condition and is associated with an increased risk of stroke, cardiac and all-cause mortality [1]. Incidence of AF increases substantially with age, from approximately 1.1 per 1000 person-years in those aged 55 to 59 years to 20.7 per 1000 person-years in those aged 80–84 years [2]. Treatment with oral anticoagulants (OACs) is highly effective in reducing the risk of stroke, and effectiveness is maintained in older age groups [3]. National and international guidelines recommend treatment with OACs for patients with risk factors for stroke [4, 5]; however, they have historically been underused in older patients despite advancing age being a significant risk factor for stroke [3].

The first direct oral anticoagulant (DOAC), dabigatran, was marketed across the European Union and the United Kingdom (UK) in 2008; however, it was not licensed for stroke prevention in AF until 2011. Three additional DOACs have since been licensed for this indication, rivaroxaban, apixaban and edoxaban, and these are recommended in both international and national guidelines as an alternative to warfarin and other vitamin K antagonists [4–6]. The National Institute for Health and Care Excellence (NICE) produced favourable technology appraisals for all four DOACs between 2012 and 2015 [7–10], meaning that the National Health Service (NHS) was required to fund the DOACs for stroke prevention from mid-2012 onwards.

Overall, the rate of OAC initiation has increased by 58% since the DOACs were introduced [11]. It is not known if the rates of OAC prescribing have changed for older people (aged ≥ 75 years) or whether patient demographics, comorbidities or concomitant medication that may have traditionally led to under-prescribing of warfarin in this group continue to affect the chance of receiving an OAC in the post-DOAC era.

This study aims to characterise how the introduction of DOACs has affected anticoagulant prescribing to people aged ≥ 75 years in UK general practice and answer the following questions:

1. Has the incidence and prevalence of OAC prescribing to people aged ≥ 75 years changed in the period prior to the introduction of DOACs (2003–2007), between the time DOACs were introduced and the time they were recommended by NICE (2008-2012), and following NICE recommendation (2013–2017)?
2. How do older people switch between different OACs?
3. Which patient characteristics and co-morbidities affect the chance of being prescribed an OAC and has this changed since the introduction of DOACs?
4. How does persistence with therapy differ between different OACs?

## Methods

This retrospective cohort study examined trends in OAC prescribing for older people with AF in UK general practice before and after the introduction of DOACs, using routinely collected healthcare data. Detailed methods for this study have been published previously [12].

The data were sourced from the Clinical Practice Research Datalink (CPRD) GOLD database. The CPRD contains anonymised medical records and prescribing data from general practitioners in primary care. It contains data for around 7% of the UK population and is representative in age, sex and ethnicity [13]. Data are coded in the CPRD using Read codes [13], these codes were used to identify eligible patients and to identify sociodemographic, medical diagnoses and other clinical and test data of interest.

The study period was 1st January 2003 to 27th December 2017. The source population of patients consisted of all patients in the CPRD who, before or during the study period, had one or more Read codes for AF on separate dates, or one Read code for AF plus one or more Read codes indicating evidence of AF (e.g., referral to a cardiologist). Patients from this source population could enter the study cohort at the latest of the following dates:

- Start of the study period
- First AF diagnosis
- 75th birthday
- The earliest date on which the patient had contributed a year of research standard data (defined as the point at which the general practice submitted 1 year of data meeting the CPRD’s data standard following patient registration)

Patients were censored if they left the general practice, the general practice stopped contributing data, at their date of death, at the end of the study, or when they were prescribed an OAC (for all analyses except prevalence and switching). Patients who never received an OAC prescription but were found to have more than one Read code suggesting they were started on an OAC during the study (e.g., oral anticoagulant prescribed by the third party) were censored at the date of the first Read code. Patients who started on, or were switched to a non-warfarin vitamin K antagonist during the study, were censored on the day before the date of the prescription.

Patients were excluded if they had a Read code for venous thromboembolism in 6 months preceding their first OAC prescription or if they had a Read code for total hip replacement (THR) in 6 weeks prior to a single OAC prescription as the OAC may not have been prescribed for stroke prevention in AF. Patients were included despite having THR if they received more than one consecutive OAC prescription as the normal duration for thromboprophylaxis following THR is 6 weeks. OACs may also be prescribed for 2 weeks following a total knee replacement, but these patients were not excluded as the OAC for this indication would normally be prescribed by the hospital and not in primary care. It was noted during cohort creation that a number of patients were prescribed a non-warfarin vitamin K antagonist in the year prior to study entry; there were also a number of patients with Read codes suggesting that they were prescribed an OAC (e.g., anticoagulant prescribed by the third party) but the patients were not issued any OAC prescriptions. The presence of these patients had not been anticipated when the protocol was developed but the decision was made to exclude them.

The study was divided into three time periods:

- Period 1: 2003 to 2007 (prior to the introduction of DOACs)
- Period 2: 2008 to 2012 (during the period between the introduction of DOACs and the time they were recommended by NICE)
- Period 3: 2013 to 2017 (following the publication of the NICE technology appraisals recommending DOACs as an option for stroke prevention in AF)

Exposure status was defined at study entry as ‘no OAC’, ‘incident OAC’ or ‘prevalent OAC’. Patients with a year free of OAC before study entry and no OAC prescribed during the study were classed as ‘no OAC’, those with a year free of OAC who were started on one during the study period were classed as ‘incident OAC’ and those who received an OAC in the year prior to study entry were classified as ‘prevalent OAC’. Incident patients were further stratified at the start of each time period to either ‘no OAC’, warfarin or DOAC. Patients could contribute data to more than one group and more than one time period.

Prescriptions for the OACs of interest (warfarin, dabigatran, rivaroxaban, apixaban and edoxaban) were identified during the study period and in the year prior to study entry. For incident OAC patients, the first OAC prescription recorded during the study period was defined as the index OAC. Prescriptions were mapped using the quantity prescribed and the licensed number of doses per day for the DOACs (one for rivaroxaban and edoxaban and two for apixaban and dabigatran). Gaps of ≤ 60 days were filled, and patients were classed as having discontinued the DOAC if there was a period of more than 60 days between the end of one prescription and the start of the next. Dosing information for warfarin is not routinely recorded in the CPRD so the duration of exposure was estimated using a combination of prescription data, gaps between prescriptions and international normalised ratio (INR) test results. The algorithm was further improved by including Read codes suggesting that warfarin therapy had either continued or stopped (e.g., warfarin contraindicated).

Yearly incidence of OAC prescribing was calculated overall (stratified by age and sex) and for each specific OAC. People were considered ‘at risk’ until their index OAC prescription. The numerator was the number of incident patients, and the denominator was the person-time at risk for non-exposed persons. For the person-time at risk, the denominator was truncated at the index date. Point prevalence was calculated at the mid-point of each year. The number of people with an OAC prescription spanning the mid-point of the year was included in the numerator and the total number of people in the cohort at the same time point was included in the denominator. The numbers and percentages of patients switching between OACs were calculated separately for patients in the incident and prevalent groups. Poisson regression was used to model the change in prescribing of OACs over time with covariates for year of study, the availability of DOACs and the number of OACs available each year used in the model [14]. To evaluate trends in prescribing, the non-parametric test for trend was used and the coefficients for individual Poisson regressions of prescribing of warfarin and prescribing of DOACs over time were compared using the Hausman test.

To compare the characteristics of patients newly started on an OAC during each period, demographics and comorbidities were collected for both the ‘no OAC’ and ‘incident OAC’ groups. For each period, patients were assigned to either the ‘OAC’ or ‘no OAC’ group. For those in the ‘no OAC’ group, demographic and comorbidity details were obtained from data accumulated prior to the end of the time period (or the study exit date if this occurred sooner). Stroke risk was calculated using the CHA_2_DS_2_-VASc score (one point assigned for congestive heart failure, hypertension, diabetes mellitus, vascular disease or female sex; two points for age ≥ 75 years and stroke, transient ischaemic attack or thromboembolism) [15]. Bleeding risk was calculated using a modified HAS-BLED score (one point for hypertension, renal or liver disease, stroke, major bleeding or predisposition to bleeding, age > 65 years, medication use predisposing to bleeding or alcohol misuse. Labile INR was omitted as this data is not reliably recorded in the CPRD) [16]. For patients started on an OAC during the period, details were obtained from data accumulated until the index date. Data on concomitant medication was collected for 3 months prior to the end of the period or the study exit date (if this was sooner) for the ‘no OAC’ group and for 3 months prior to the index date for patients in the ‘incident OAC group’. Patients started on an OAC were then censored so could not contribute data to later periods. To account for the increase in the incidence of OAC prescribing over the three time periods, the risk of starting an OAC was calculated separately in each period for patients with and without each comorbidity. These risks were used to calculate the risk difference and 95% confidence interval of being newly prescribed an OAC if the comorbidity was present in each time period.

Missing data for weight, body mass index, smoking and alcohol were investigated using logistic regression to ascertain whether other variables (age, sex or comorbidities) predicted whether these data were missing at random. Missing data were found to be significantly associated with a number of other variables so not missing at random, for this reason, we decided it was not appropriate to impute the missing data. We did not exclude patients with missing data; they were compared with the baseline group and these data were reported (e.g., the proportion of patients prescribed an OAC where the smoking status was unknown (missing data) compared with the baseline group non-smokers).

Logistic regression was used to ascertain which demographics and co-morbidities were associated with starting a DOAC compared with warfarin in period 3. Characteristics and co-morbidities that were strongly associated with prescribing of a DOAC over warfarin or vice-versa were added to a multivariable model in a forward step-wise approach, those that remained significant and could also have a plausible mechanism to affect time on OAC therapy were designated as potential confounders for the persistence analysis.

To describe persistence with DOACs compared with warfarin, a survival analysis was conducted using Cox-proportional hazards stratified by DOAC type. Both unadjusted and adjusted estimates were calculated, adjusting for age, sex and covariates identified as potential confounders above. The proportional hazards assumption was assessed by examining Schoenfeld residuals. The proportional hazards assumption was violated, so the follow-up was partitioned by year and further examination of the Schoenfeld residuals then showed the proportionality assumption to be met.

All analyses were conducted in Stata version 16.

## Results

**Fig. 1:**
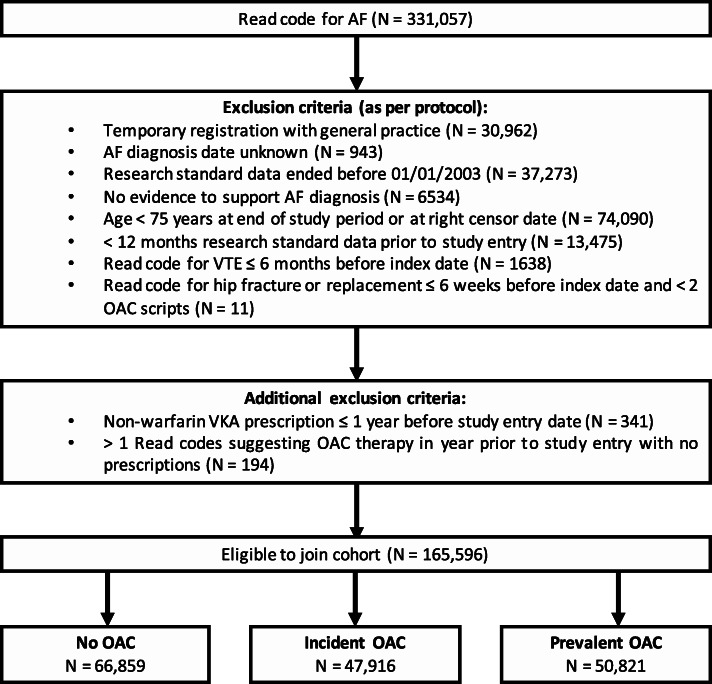
Flow chart of cohort development and exclusions at each stage

The final cohort consisted of 165,596 patients. Figure 1 illustrates the process of cohort development and details exclusions at each stage.

In total, there were 66,859 patients who were never prescribed an OAC, 47,916 patients were ‘incident OAC users’ who were newly prescribed an OAC during the study, and 50,821 patients who were prescribed an OAC in the year preceding study entry so were included in the ‘prevalent OAC user’ group.

Table 1 shows selected patient demographics, co-morbidities and co-medication at baseline for each group (see Additional file 1: Table S1 for the full table). Patient demographics were broadly similar in each of the three groups. The mean CHA_2_DS_2_-VASC (no OAC, 4.2 (SD 1.4), incident OAC 4.1 (SD 1.3), prevalent OAC 4.5 (SD 1.5)) and HAS-BLED scores (no OAC, 3.3 (SD 1.2), incident OAC 3.4 (SD 1.3) and prevalent OAC 3.0 (SD 1.2)) were similar for each group suggesting similar stroke and bleeding risks. Patients in the no OAC group were older (median 84 years, IQR 79–89). There were more females in the group that were not prescribed an OAC (no OAC, 60%; incident OAC, 51%; prevalent OAC 48%). The no OAC group had a lower median body weight but also had the largest proportion of missing data (no OAC 69kg, IQR 59–80kg; incident OAC 75kg, IQR 65–86kg; prevalent OAC 76kg, IQR 66–88kg). The proportion of missing data for smoking, alcohol status and weight was substantially higher in the no OAC group, than either the incident or prevalent OAC groups (see Additional file 1: Table S1) making comparisons of the effect of these characteristics on OAC use subject to bias. Investigation suggested that missing data was not missing at random and that both age and sex influenced whether these data were missing, so they were not imputed. We have therefore not reported these characteristics, but data are presented in Additional file 1: Table S1 for information.

**Table 1:** Characteristics at baseline of patients aged ≥ 75 years with atrial fibrillation included in the CPRD between 2003 and 2017

	No OAC (*n* = 66,859)	Incident OAC (*n* = 47,916)	Prevalent OAC (*n* = 50,821)
Median age in years (IQR)	84 (79–89)	80 (77–84)	77 (75–82)
75–79	19,417 (29.0)	22,001 (45.9)	31,543 (62.1)
80–84	16,999 (25.4)	15,079 (31.5)	10,741 (21.1)
85–89	16,442 (24.6)	8161 (17.0)	6011 (11.8)
90+	14,001 (20.9)	2675 (5.6)	2526 (5.0)
Sex (female)	40,029 (59.9)	24,482 (51.1)	24,461 (48.1)
Median weight in kg (IQR)	69 (59–80)	75 (65–86)	76 (66–88)
Median body mass index (IQR)	25.3 (22.4–28.6)	26.9 (24.1–30.2)	26.8 (23.9–30.2)
Mean CHA_2_DS_2_-VASc score (SD)	4.2 (1.4)	4.1 (1.3)	4.5 (1.5)
Mean HAS-BLED score (SD)	3.3 (1.2)	3.4 (1.3)	3.0 (1.2)
Co-morbidities at study entry			
Heart failure	12,415 (18.6)	5116 (10.7)	13,307 (26.2)
Diabetes mellitus	10,158 (15.2)	7829 (16.3)	10,053 (19.8)
Hypertension	38,630 (57.8)	31,845 (66.5)	31,123 (61.2)
Ischaemic stroke, transient ischaemic attack, or thromboembolism	13,084 (19.6)	7805 (16.3)	14,708 (28.9)
Coronary artery disease	17,439 (26.1)	12,248 (25.6)	15,566 (30.6)
Peripheral vascular disease	6349 (9.5)	4194 (8.8)	6568 (12.9)
Fragility fracture	13,453 (20.1)	7198 (15.0)	7097 (14.0)
Heart valve replacement or mitral stenosis	738 (1.1)	626 (1.3)	3072 (6.0)
Dementia	6649 (9.9)	909 (1.9)	1998 (3.9)
Chronic renal impairment	13,141 (19.7)	11,681 (24.4)	10,784 (21.2)
Acute kidney injury	1214 (1.8)	2969 (6.2)	2298 (4.5)
Previous bleed (any)	22,500 (33.7)	16,857 (35.2)	19,333 (38.0)
Major bleed	2667 (4.0)	1169 (2.4)	1728 (3.4)
Clinically relevant non-major bleed	20,825 (31.1)	16,161 (33.7)	18,349 (36.1)
Intracranial haemorrhage	1340 (2.0)	378 (0.8)	649 (1.3)
Gastrointestinal bleeding	8172 (12.2)	5939 (12.4)	6086 (12.0)
Other bleed	22,500 (33.7)	16,857 (35.2)	19,333 (38.0)
One or more falls in past year	6548 (9.8)	2258 (4.7)	2682 (5.3)
Mean number of GP encounters in year prior to study entry (SD)	12.5 (10.9)	11.7 (9.3)	17.6 (13.5)
Medication prescribed within 3 months of study entry			
Antiplatelets	38,696 (57.9)	24,782 (51.7)	6654 (13.1)
Corticosteroids	4883 (7.3)	3674 (7.7)	3159 (6.2)
Non-steroidal anti-inflammatories	4282 (6.4)	3258 (6.8)	1259 (2.5)
Proton pump inhibitor or H_2_ receptor antagonist	21,170 (31.7)	15,447 (32.2)	14,810 (29.1)
Selective serotonin reuptake inhibitors	5752 (8.6)	2329 (4.9)	3465 (6.8)
Statins	19,113 (28.6)	20,627 (43.0)	23,679 (46.6)

Results are presented as number (%) of patients, median (interquartile range) or mean (standard deviation). *GP* General practice, *CHA*_*2*_*DS*_*2*_*-VASc* Stroke risk score, *HAS-BLED* Bleeding risk score

The presence of most comorbidities was highest in the prevalent OAC group. Hypertension (66.5%) and renal disease (6.2% for acute kidney injury (AKI); 24.4% for chronic impairment) were most common in the incident OAC group. Dementia (9.9%) and fragility fracture (20.1%) were most common in the no OAC group. Major bleeds and intracranial haemorrhage were also more common in the no OAC group (4% for major bleed; 2% for intracranial haemorrhage). The mean number of encounters with general practice in the year preceding study entry was highest in the prevalent OAC group but similar in the incident and no OAC groups (prevalent OAC, 17.6 (SD 13.50); no OAC, 12.5 (SD 10.9); and incident OAC 11.7 (SD 9.3)).

### Incidence and prevalence of oral anticoagulant prescribing

**Fig. 2:**
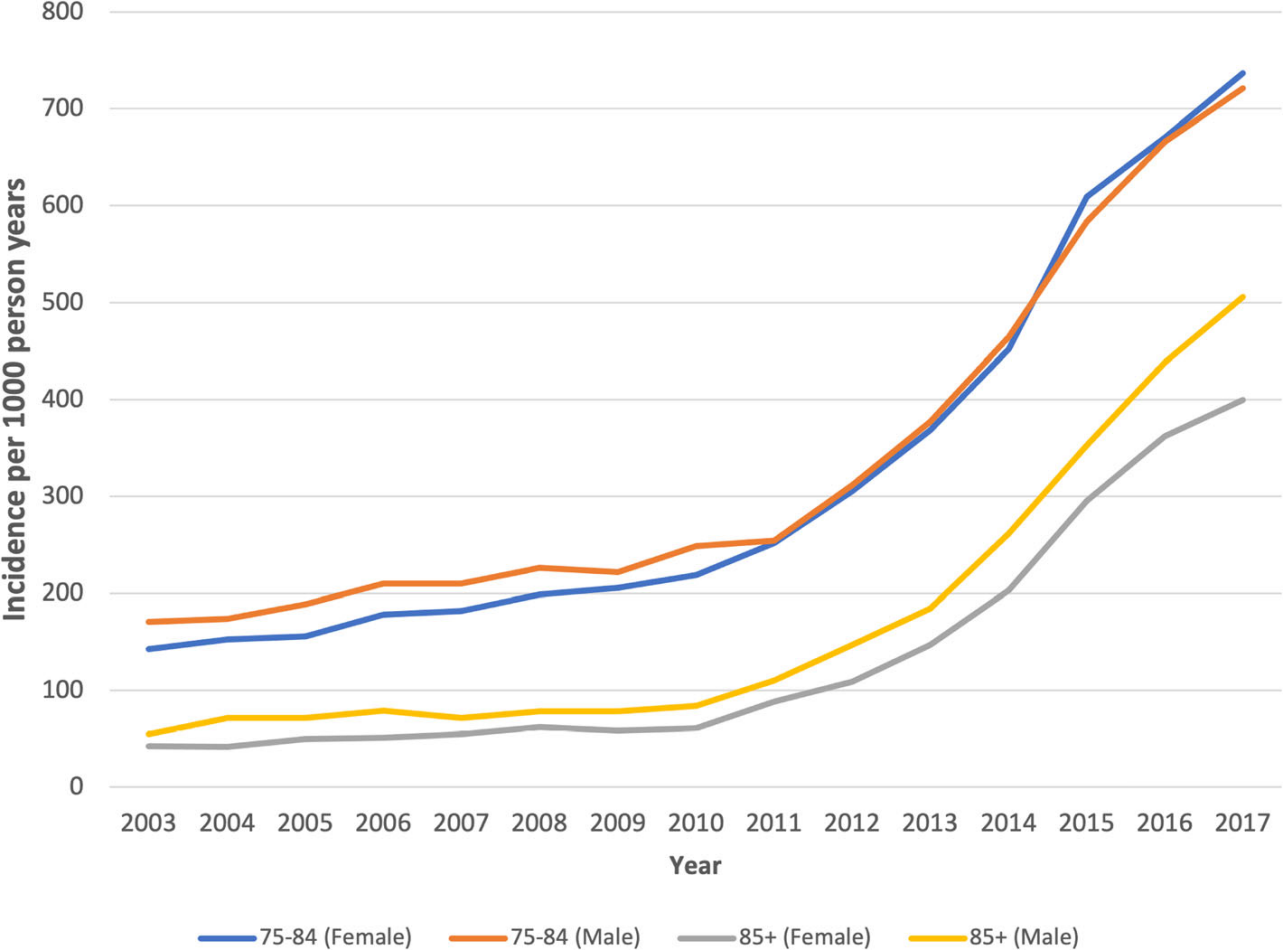
Incidence of oral anticoagulant prescribing to patients aged ≥ 75 years with a diagnosis of atrial fibrillation by year stratified by age and sex

The incidence of OAC prescribing increased from 111 per 1000 person-years to 587 per 1000 person-years between 2003 and 2017. This upward trend was also seen when incidence was stratified by age band and sex (see Fig. 2). The incidence of OAC prescribing in the pre-DOAC era (2003–2007) was 122.6 per 1000 person-years. This increased to 164.2 per 1000 person-years in the era when DOACs were available but not yet recommended by NICE, then more than doubled to 387 per 1000 person-years in the era following NICE recommendation (2013–2017). Stepped Poisson regression (see Additional file 1: Fig. S1) highlighted this change in prescribing of DOACs compared with warfarin with a 6.5% increase each year (Incidence rate ratio (IRR): 1.065, 95% CI 1.06 −1.07) and a more than doubling prescribing of DOACs compared with warfarin (IRR: 2.25, 95%CI 2.14–2.37). Non-parametric test for trend of prescribing of warfarin reported a non-significant value (*p*=0.89) but prescribing of DOACs showed a significant increasing trend (*p*=0.008). However, given that the warfarin prescribing was not linear, Poisson regression was also used to separately model the prescribing of warfarin and prescribing of DOACS; the model coefficients were compared and these were significantly different (*p*<0.001).

**Fig. 3:**
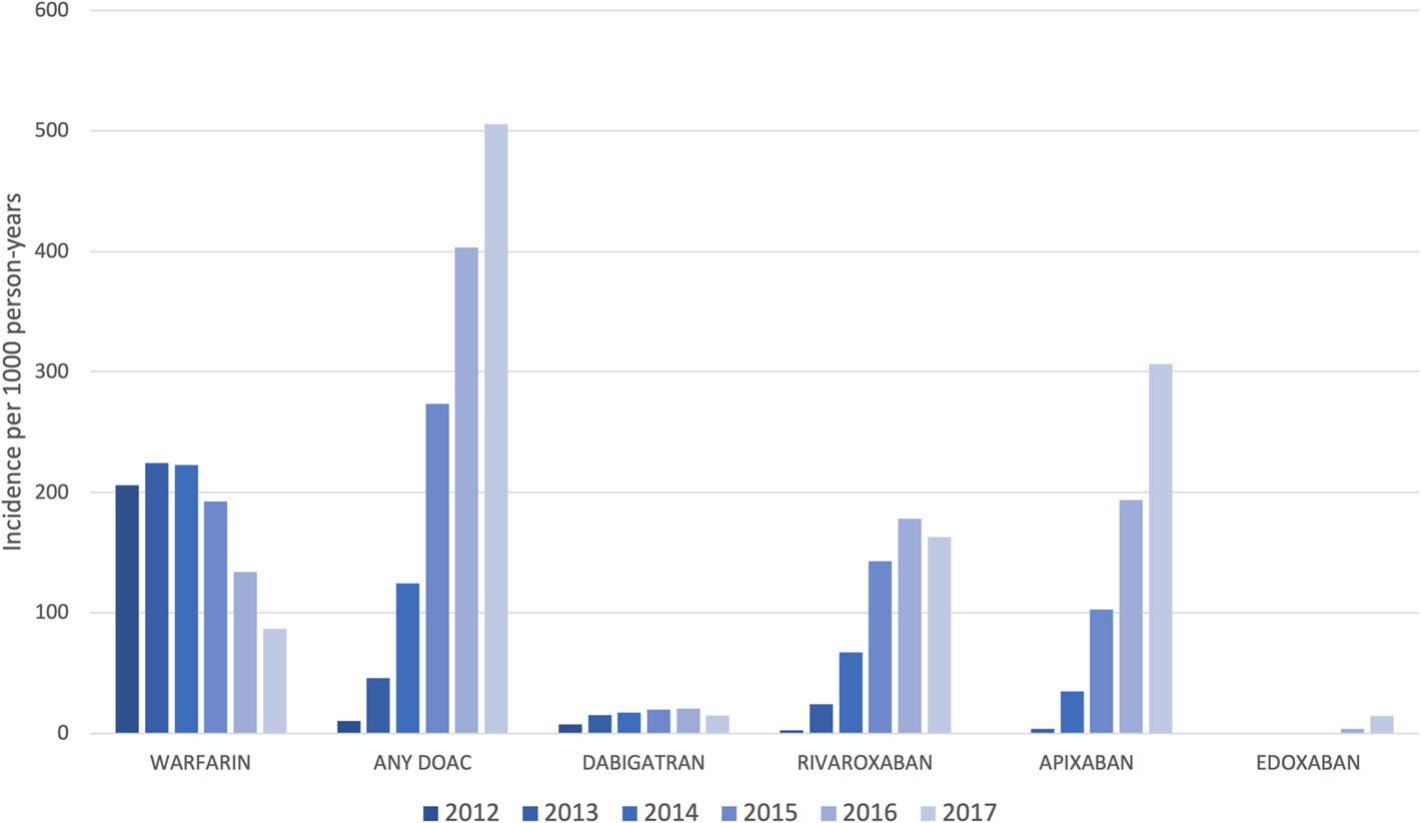
Incidence of oral anticoagulant prescribing to patients aged ≥ 75 years with a diagnosis of atrial fibrillation by year stratified by OAC type in the years 2012 to 2017

From 2012 to 2017, i.e., when both DOACs and warfarin were marketed, the incidence of warfarin prescribing decreased and DOAC prescribing increased rapidly (Fig. 3). Apixaban was the most commonly prescribed DOAC followed by rivaroxaban then dabigatran. Edoxaban was licensed toward the end of the study period in 2015 and therefore had the lowest incidence of prescribing.

The prevalence of OAC prescribing to patients aged ≥ 75 years with a diagnosis of AF increased every year. In 2003, the point prevalence of OAC prescribing was 37.6% increasing to 75% in 2017. The point prevalence of warfarin prescribing to this group decreased from 54.8% in 2013 to 44% in 2017 whilst the prevalence of DOAC prescribing increased from 1.9% in 2013 to 31.1% in 2017.

### Switching between anticoagulants

Switching was calculated separately for those in the incident and prevalent groups. In the incident group, there were 5467 switches in 4566 patients and in the prevalent group, there were 821 switches in 621 patients. The majority of patients in both the incident (86.6%) and prevalent groups (79.2%) switched only once. A small proportion of patients (3.8% of the incident group and 6% of the prevalent group) had 3 or more switches during the study. The most common switch type was warfarin to DOAC, and the least common switch was DOAC to warfarin. Table 2 describes the number of switches and the proportion of each switch type in each group.

**Table 2:** Number (percentage of total switches in group) of each switch type in patients who are newly started on an OAC during the study (incident group) or who entered the study on an OAC (prevalent group)

	*Incident* *(N = 5467 switches)*	*Prevalent* *(N = 821 switches)*
*Warfarin to DOAC*	*3883 (71)*	*343 (41.8)*
*DOAC to warfarin*	*468 (8.6)*	*228 (27.8)*
*DOAC to DOAC*	*1116 (20.4)*	*250 (30.4)*

Switches were categorised as direct (no gap between OACs) or indirect (a period of time unexposed to any OAC before starting the next one). In both the incident and prevalent groups, the first switch was commonly a direct switch (73% and 78%, respectively). For direct switches, the median time on the index OAC prior to switching was 662 days (IQR 164–1451 days) for the incident group. For indirect switches, the median time on the index OAC was 251 days (IQR 54–869 days) in the incident group. The median unexposed gap between stopping the index OAC and starting the next OAC was 230 days (IQR 40–743 days) in the incident group and 101 days (IQR 35–427 days) in the prevalent group.

### Comparison of oral anticoagulant prescribing by sociodemographics, co-morbidities and co-medication

**Fig. 4:**
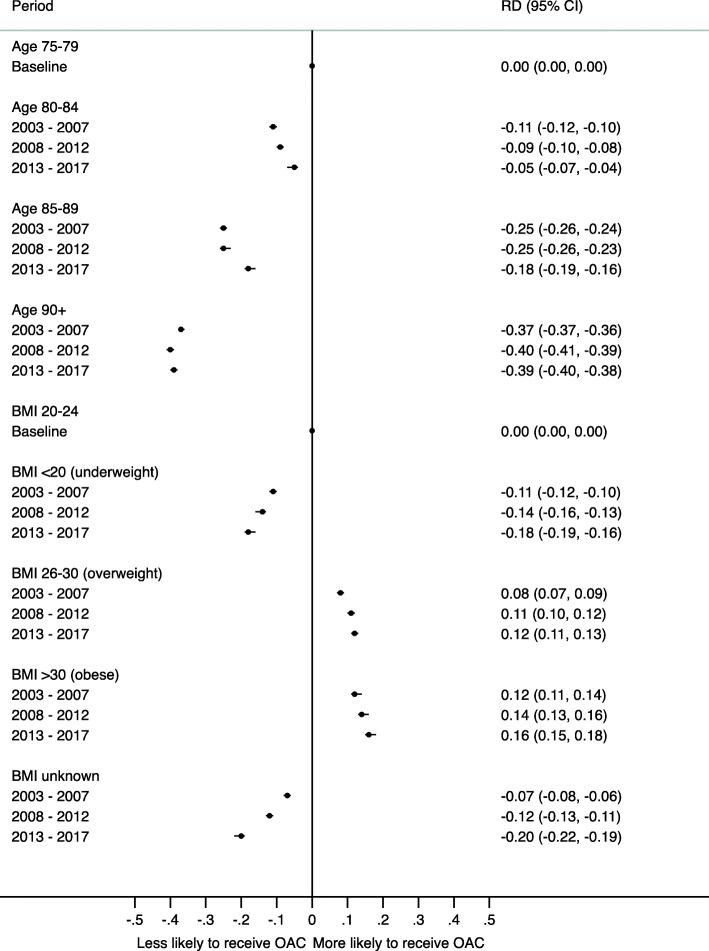
Risk difference (RD) of being prescribed an OAC for patients aged ≥ 75 years with AF in different demographics by period (2003–2007 = Pre-DOAC; 2008–2012 = DOACs available but not recommended by NICE; 2013–2017 = post-DOAC recommendation by NICE)

Figure 4 shows the risk difference (RD) in prescribing of OACs to patients with different demographics. Patients in the older age groups were less likely to receive an OAC than younger patients. Patients aged ≥ 90 years were 40% less likely to receive an OAC than those in the 75–79 year age group, and this difference was maintained in each time period. Women were slightly less likely to receive an OAC than men (risk difference around 6% in each period). Risk differences were also calculated based on smoking and alcohol status, but results are limited due to missing data (see Additional file 1: Fig S2-S3).

OAC prescribing was similar for CHA_2_DS_2_-VASC scores less than six (equating to an estimated annual stroke risk of < 9.7% [17]), but patients with a score greater than six (those at the highest stroke risk) were less likely to receive an OAC in all periods compared with those with a score of two or three (period 1: RD −0.06, 95% CI −0.07 to −0.05; period 2: RD −0.04, 95% CI −0.05 to −0.03; period 3: RD −0.06, 95% CI −0.07 to −0.05). Higher HAS-BLED scores (indicating an increased bleeding risk) were associated with a lower proportion of patients prescribed an OAC in all periods, but the difference was greatest in period one for HAS-BLED scores of 5–8 compared with a score of 0–2 (period 1: RD −0.12, 95% CI −0.13 to −0.11; period 2: −0.05, 95% CI −0.06 to −0.04; period 3: RD −0.03, 95% CI −0.04 to −0.01). Full results are shown in Additional file 1: Fig. S4.

**Fig. 5:**
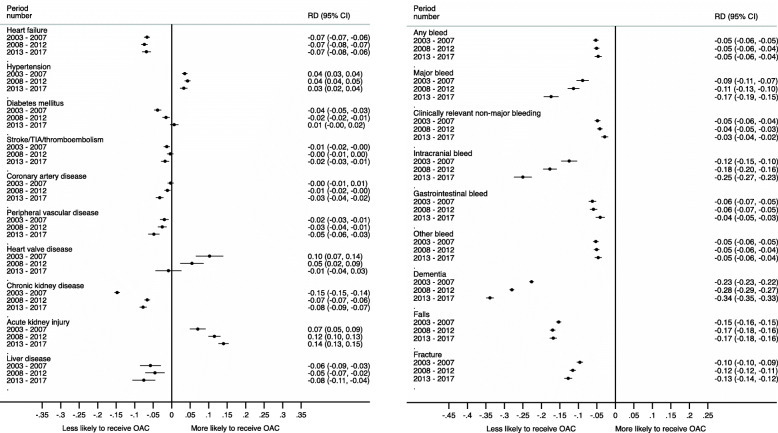
Difference in the risk of being started on an OAC in each period (2003–2007 = Pre-DOAC; 2008–2012 = DOACs available but not recommended by NICE; 2013–2017 = post-DOAC recommendation by NICE) for patients aged ≥ 75 years with AF if comorbidity present compared with those without the comorbidity

Figure 5 shows the difference in OAC prescribing when a co-morbidity is present to when the co-morbidity is not present. The co-morbidities with the largest impact on prescribing were bleeds, dementia, falls and fractures. A history of major bleeding significantly reduced OAC prescribing (period 1: RD −0.09, 95% CI −0.11 to −0.07; period 2: RD −0.11, 95% CI −0.13 to −0.1; period 3: RD −0.17, 95% CI −0.19 to −0.15). Intracranial bleeding had the greatest impact on OAC prescribing and the impact increased over time (period 1: RD −0.12, 95% CI −0.15 to −0.10; period 2: RD −0.18, 95% CI −0.2 to −0.16; period 3: RD −0.25, 95% CI −0.27 to −0.23). The impact of dementia on OAC prescribing also increased over time, in period 1 patients with dementia were 23% less likely to receive an OAC than those without and this increased to 34% in period 3 (period 1: RD −0.23, 95% CI −0.23 to −0.22; period 2: RD −0.28, 95% CI −0.29 to −0.27; period 3: RD −0.34, 95% CI −0.35 to −0.33). A history of falls reduced OAC prescribing by 17% in all periods and fracture by 12%.

Patients with a prosthetic heart valve replacement or moderate-to-severe mitral regurgitation (formally known as ‘valvular AF’) were 5–10% more likely to be prescribed an OAC in periods 1 and 2 (pre-DOAC) than those without these co-morbidites; however, in period 3, there was no difference in OAC prescribing between those with and without these conditions (period 1: RD 0.1, 95% CI 0.07 to 0.14; period 2: RD 0.05, 95% CI 0.02 to 0.09; period 3: RD −0.01, 95% CI −0.04 to 0.03).

**Fig. 6:**
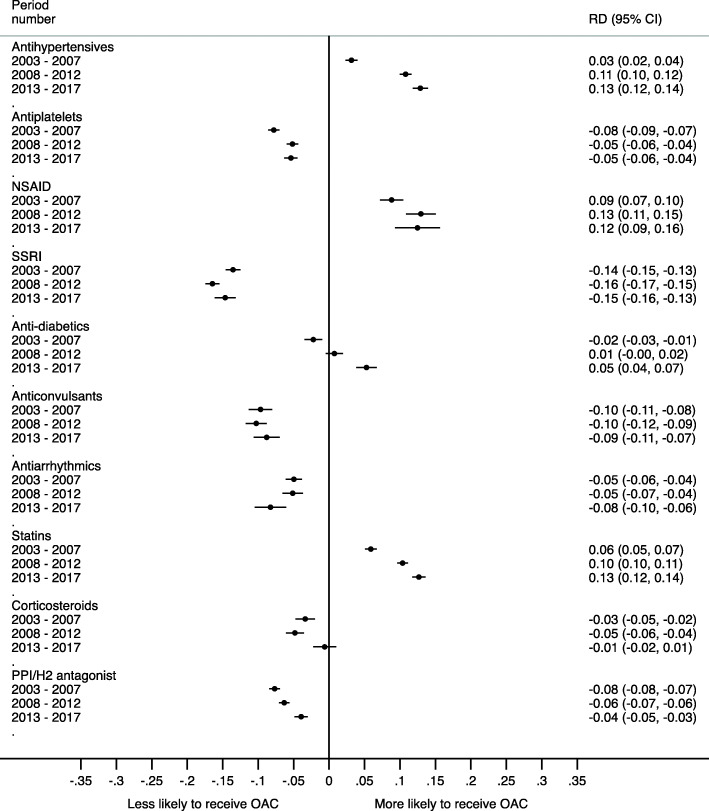
Difference in risk of being started on an OAC in each period (2003–2007 = Pre-DOAC; 2008–2012 = DOACs available but not recommended by NICE; 2013–2017 = post-DOAC) for patients aged ≥ 75 years with AF if other medication type present compared with those without the medication

Patients prescribed antihypertensives, NSAIDs or statins were over 10% more likely to receive an OAC. Patients prescribed SSRIs or anticonvulsants were 10–14% less likely to receive an OAC. Figure 6 shows the full results.

### Persistence with oral anticoagulants

Results from the univariate and multivariable logistic regression models are shown in Additional file 1: Tables S2-S3. Factors identified as potential confounders and added to the persistence analysis were dementia, age, intracranial haemorrhage, valve disease, a fall in the year preceding OAC therapy, acute kidney injury, a history of falls, a history of fracture, a history of stroke, TIA or thromboembolism.

**Fig. 7:**
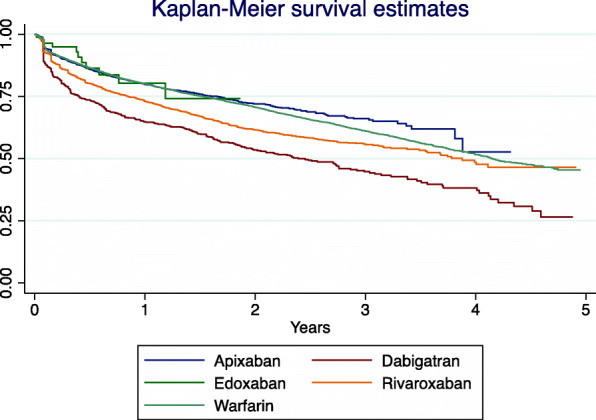
Unadjusted Kaplan-Meier survival curves for period 3 (2013–2017) illustrating the time to failure on the index OAC for patients aged ≥ 75 with AF newly started on an OAC during the study (incident group)

Figure 7 shows the unadjusted Kaplan-Meier survival estimates in period 3. Failure (i.e., time to treatment discontinuation) was fastest with dabigatran followed by rivaroxaban, warfarin and apixaban had similar time to failure.

The unadjusted Cox model suggested that persistence with DOACs was lower than with warfarin as patients started on DOACs were more likely to stop therapy (HR 1.22, 95% CI 1.15–1.28). This result remained after adjusting for confounders (HR 1.16, 95% CI 1.11–1.23). However, the assumption of proportional hazards was violated with the basic Cox model so a time dependent effect was used to improve the modelling of the baseline hazard function by individual year of study. This showed that in the first and second years of treatment, patients were more likely to stop a DOAC than warfarin (0–1 year: HR 1.25, 95% CI 1.17–1.33; 1–2 years: HR 1.12, 95% CI 0.99–1.28), but from the third year onwards, patients were more likely to persist with DOACs than warfarin (HR 0.75, 95% CI 0.63–0.89). Results when comparing individual DOACs to warfarin in the first year of treatment (adjusted model) showed heterogeneity. Persistence was lower with dabigatran (HR 1.99, 95% CI 1.76–2.25) and rivaroxaban (HR 1.37, 95% CI 1.27–1.48) than warfarin, but apixaban (HR 0.96, 95% CI 0.88–1.05) was similar to warfarin. Persistence with edoxaban past the first year was not calculated due to low patient numbers.

In the first year of treatment, persistence was higher with apixaban than either dabigatran (HR 0.49, 95% CI 0.43 to 0.56) or rivaroxaban (HR 0.7, 95% CI 0.64 to 0.77). Persistence with rivaroxaban was also higher than dabigatran (HR 0.7, 95% CI 0.61 to 0.8).

## Discussion

This cohort study is the first to provide a detailed overview of anticoagulation prescribing in older people with AF in the UK, and how use has changed since DOACs were recommended by NICE. We found that both the incidence and prevalence of OAC prescribing to people aged ≥ 75 years increased over time, but that the increase accelerated following the introduction of DOACs. Whilst OAC prescribing increased overall, there remained significant differences in prescribing to patients with certain demographics or co-morbidities. Stroke risk (as calculated by the CHA_2_DS_2_-VASc score) had little effect on whether an OAC was prescribed, but older age, dementia, a history of falls, fracture or a previous bleed significantly reduced the likelihood of being prescribed an OAC. Persistence with DOACs is shorter than warfarin in the first year, but from the third year of therapy this trend is reversed, and patients persist longer with DOACs than with warfarin.

### Incidence and prevalence of oral anticoagulant prescribing to older people has increased but is still lower in the oldest old

Numerous studies have confirmed an increase in OAC prescribing over the last decade in the UK for prevention of stroke in AF in the general population [11, 18–20] and to older people [21]. This change has also been observed at practice level, with general practices with a higher ratio of older people and a higher prevalence of AF more likely to prescribe DOACs [22]; however, there is a risk of ecological fallacy if wishing to extrapolate this practice level data to prescribing to individuals. Whilst some of the increase in prescribing of OACs may be due to the introduction of DOACs, it may also be attributed to the introduction of Quality Outcome Frameworks for AF which incentivised the diagnosis of AF and prescribing of anticoagulants [23, 24]. The increase in prevalence of OAC prescribing to older people has also been seen in other countries [25, 26]. The general increase in prescribing of OACs to patients aged ≥75 years is promising and suggests greater adherence to NICE and European Society of Cardiology (ESC) guidelines [4, 5]; however, prescribing to the oldest old is still significantly lower than for younger patients. Our study and others show that compared with patients aged 75–79 years, patients aged ≥85 years are up to 45% less likely to receive an OAC, and this association remains when other factors such as comorbidities are adjusted for [27–31]. Terminal illness or palliative care may influence OAC prescribing at all ages, but particularly in the oldest old, however, the number of patients in our cohort with a Read code suggesting a palliative diagnosis at study entry was very small (*n* = 1686, 1% of total cohort).

### Co-morbidities reduce OAC prescribing even when they are not a contraindication to therapy

Dementia/cognitive impairment [25, 27], a history of falls and bleeding [27, 29, 31] have been shown in previous studies to reduce OAC prescribing. Prior stroke has been associated with increased OAC prescribing [29, 31] but had no effect in our study. This finding is unexpected and may be due to other factors such as age or other comorbidities which were not adjusted for in our univariate model. Other studies have found that OAC prescribing is not significantly affected by contraindications to therapy such as major bleeding or haemorrhagic stroke [32], but this may be due to the fact that these so-called ‘contraindications’ are time dependent and a history of one of these contraindications does not necessarily preclude treatment for life. It is unsurprising that we found prior bleeds, and in particular major bleeds and intracranial haemorrhage to be associated with reduced OAC prescribing. Where serious bleeds have occurred, the risk of further bleeding needs to be balanced carefully with the risk of stroke.

Despite NICE guidance specifically recommending that anticoagulation not be withheld solely because a person is at risk of falls [5], this was not seen in our study. The reduction in OAC prescribing to people with falls remained the same in all three periods (spanning the time pre- and post- the 2014 NICE publication). There is strong evidence that patients on warfarin would have to fall around 300 times a year for the risk of bleeding to outweigh the benefit of stroke prevention [33, 34]. Studies of DOACs also suggest that patients are not at higher risk of brain injury following low level falls [35]; however, falling is still often cited by prescribers as a reason for avoiding anticoagulation [36].

Dementia is not a contraindication to anticoagulant therapy, but historically, it may not have been practical to prescribe warfarin to patients with cognitive impairment due to the complex dosing regimens. DOACs have a simpler dosing regime and can be added to compliance aids, but the gap in OAC prescribing to those with dementia compared with those without has actually increased since the introduction of DOACs. Comorbidities such as AF are common in older people with dementia, but they are often undertreated [37]. Whilst there is strong evidence that anticoagulation significantly reduces stroke risk in older people, few studies have investigated outcomes in patients with dementia [38], so it may be difficult for prescribers to weigh up the risk-benefit ratio in this group.

### Persistence with oral anticoagulation varies substantially between studies

Studies comparing persistence with warfarin and DOACs have yielded conflicting results. Persistence is defined as the time from medication initiation to discontinuation. Where persistence with medications is compared, hazard or odds ratios are reported for the time to discontinuation. A ratio greater than one indicates that the medication of interest (in this case DOACs) is discontinued sooner than the comparator (i.e., warfarin). Previous studies from the USA and Italy have found persistence with dabigatran [39] and all DOACs [40, 41] to be better than warfarin after 12 months of treatment. A UK study found that persistence with dabigatran was lower than warfarin (HR 1.24, 95% CI 1.08–1.42) but higher with rivaroxaban (HR 0.85, 95% CI 0.77–0.93) and apixaban (HR 0.53, 95% CI 0.46–0.60) [42]. The average age of patients in this study was 74 years compared with 80 years in our study. Another more recent UK study also found persistence with DOACs to be better than warfarin, particularly in older age groups (aged 85 and over) [43], but the study consisted only of those who had attended for an influenza vaccine rather than all patients with AF as in our study. This may have led to selection bias, i.e., only including those who actively engage with healthcare. Neither of these UK studies used comprehensive prescription mapping and both assigned arbitrary end dates to prescriptions. Our study used all available prescription data, test data and clinical data to more robustly estimate exposure time and account for gaps in exposure which would not be seen using the methods described by Lund and colleagues [43]. Other European studies, however, have yielded results similar to ours. A Swedish study found persistence at 1 year to be similar between apixaban (OR 0.88, 95% CI 0.62–1.25) and warfarin but lower with dabigatran (OR 1.81, 95% CI 1.57–2.10) and rivaroxaban (OR 1.50, 95% CI 1.24–1.81) [44]. A German study found the same for persistence at 1 year for DOACs compared with warfarin: apixaban (HR 1.08, 95% CI 0.95–1.24), dabigatran (HR 1.53, 95% CI 1.40–1.68) and rivaroxaban (HR 1.21, 95% CI 1.14-1.29), although they noted that when time was partitioned by first 100 days of treatment that persistence with apixaban was lower than with warfarin [45]. These differences may be due to the methods used to calculate exposure, differences in cohort characteristics or differences in each country’s healthcare systems. Some studies used ‘proportion of days covered’ to assess persistence and assumed each prescription would last 30 days [42]. This method has been shown to work well for fixed-dose medications but may be inaccurate for medications with variable doses, such as warfarin, which could have affected the results [46]. The studies with results most similar to ours used different methods to define exposure to DOACs compared with warfarin to account for the variable warfarin dosing and used INR test results in addition to prescription data. Sensitivity analyses in these studies showed that when INR tests were not used, warfarin persistence decreased [40, 44, 45].

### Strengths and weaknesses of this study

The major strengths of this study are that we used a large and representative sample of older people in the UK and extracted data from both the time prior to and post NICE approval to describe how prescribing has changed over time. We mapped OAC prescribing rather than relying on single prescriptions which allows us to consider switching and unexposed time. We have provided a wealth of data that could be used to inform future studies as it highlights a number of potential confounders that may lead to channelling bias if not considered when comparing outcomes with warfarin and DOACs in this population. A weakness of this study is that the CPRD only contains data of prescriptions and not whether these were dispensed or taken by the patient. Ultimately, this type of study cannot account for the various reasons that prescribers use to determine whether or not to prescribe an OAC. The prescription data for warfarin held in the CPRD does not accurately define the dose taken or how long a supply would last. Periods of exposure are based on estimations from an algorithm that includes clinical and test data (INR) in addition to prescription data to estimate exposure. This provides more robust estimates than prescription data alone. Diagnostic Read codes have been shown previously to accurately identify patients with AF in the CPRD with a low rate of false positives [47]. We further strengthened case identification by only including patients that had more than one diagnostic Read code for AF or evidence to support their diagnosis such as diagnostic codes or a change in prescribing that would support the diagnosis of AF. A limitation of this study is that we did not account for patients whose AF had resolved which could be a reason for not prescribing an OAC. Only a small number of patients (5% of those in the no OAC group) had a Read code suggesting that their AF had resolved during the study so this would not have significantly changed our results had we excluded them. We did not exclude patients with thrombotic disorders which may contraindicate OAC therapy; however, we would anticipate the number of patients with clotting disorders to be small. We have used the CHA_2_DS_2_-VASc score to define stroke risk in this study, but it should be acknowledged that this score was not recommended until 2010 [15]. The CHA_2_DS_2_-VASc score replaced the CHADS_2_ score as it was found to better discriminate truly low-risk patients [15]. The guidance on when to anticoagulate has also changed over the study period: the 2006 NICE guidelines [48] recommended that those at moderate risk (as per the CHADS_2_ score) should consider aspirin or an anticoagulant whereas the 2014 NICE guideline [5] no longer recommends aspirin. We have not considered aspirin use as it is no longer recommended, and our focus was on anticoagulation.

## Conclusions

This study has shown that whilst the incidence of anticoagulant prescribing was five times higher in 2017 than 2003 in older people for stroke prevention in AF, there are still substantial differences in who is prescribed these medications based on their demographics and comorbidities. Advancing age and dementia have consistently been associated with reduced OAC prescribing by up to 40% since 2003, but neither are contraindications. Further research is needed to establish the absolute risks and benefits in these groups to enable better informed prescribing. Guidelines need to address these under-represented groups to advise when it is appropriate to offer preventative medicine such as anticoagulation but also when it should be stopped. As shown in this study, however, incorporation into guidelines (as with the recommendations about patients who fall) may not be enough. Prescribers need to be convinced that they can trust the evidence and the guidance and that under-treating their patients can have consequences as severe as those they associate with the treatment.

## Supplementary Information

**Additional file 1: TableS1.** Sociodemographics and comorbidities at baseline (extended version). **TableS2.** Univariate logistic regression model showing which characteristics and co-morbidities are associated with the prescribing of a DOAC over warfarin. **TableS3.** Factors which remained significant in the multivariate model. **FigS1.** Rate of prescribing of OACs in the AF population over time. **FigS2**. Risk difference of being prescribed an OAC by smoking status. **FigS3.** Risk difference of being prescribed an OAC by alcohol status. **FigS4.** Risk difference of being prescribed an OAC stroke and bleeding risk.

## Data Availability

The data that support the findings of this study are available from the Clinical Practice Research Datalink, but restrictions apply to the availability of these data, which were used under license for the current study, and so are not publicly available. The Read code lists used to identify AF, co-morbidities and concomitant medication are available on request from the corresponding author.

## Abbreviations

AF: Atrial fibrillation
BMI: Body mass index
CHA_2_DS_2_-VASc: Stroke risk score (one point assigned for congestive heart failure, hypertension, diabetes mellitus, vascular disease or female sex; two points for age ≥ 75 years and stroke, transient ischaemic attack or thromboembolism)
CHADS_2_: Stroke risk score (one point assigned for congestive heart failure, hypertension, age ≥ 75 years, diabetes mellitus; two points for stroke)
CI: Confidence interval
CPRD: Clinical Practice Research Datalink
DOAC: Direct oral anticoagulant
HAS-BLED: Bleeding risk score (one point for hypertension, renal or liver disease, stroke, major bleeding or predisposition to bleeding, age > 65 years, medication use predisposing to bleeding or alcohol misuse. Labile INR was omitted as this data is not reliably recorded in the CPRD)
HR: Hazard ratio
INR: International normalised ratio
IRR: Incidence rate ratio
IQR: Interquartile range
NHS: National Health Service
NICE: National Institute for Health and Care Excellence
OAC: Oral anticoagulant
OR: Odds ratio
RD: Risk difference
SD: Standard deviation
THR: Total hip replacement
UK: United Kingdom
VTE: Venous thromboembolism
