# Supplementary material for: Prescribing of direct oral anticoagulants and warfarin to older people with atrial fibrillation in UK general practice: a cohort study
Source: BMC Med. 2021 Aug 31;19:189. doi: 10.1186/s12916-021-02067-5 (PMC8406735; doi:10.1186/s12916-021-02067-5)
Supplement: Supplementary file 1 — Additional file 1: TableS1. Sociodemographics and comorbidities at baseline (extended version). TableS2. Univariate logistic regression model showing which characteristics and co-morbidities are associated with the prescribing of a DOAC over warfarin. TableS3. Factors which remained significant in the multivariate model. FigS1. Rate of prescribing of OACs in the AF population over time. FigS2. Risk difference of being prescribed an OAC by smoking status. FigS3. Risk difference of being prescribed an OAC by alcohol status. FigS4. Risk difference of being prescribed an OAC stroke and bleeding risk. [file 12916_2021_2067_MOESM1_ESM.docx]

**Additional File 1 – Supplementary results**

*Table S1. Sociodemographics and comorbidities at baseline (comorbidities recorded prior to study entry) of patients aged ≥ 75 years with atrial fibrillation included in the CPRD between 2003 and 2017 (extended version)*

|  | No OAC  (n = 66,859) | Incident OAC  (n = 47,916) | Prevalent OAC  (n = 50,821) |
| --- | --- | --- | --- |
| Median age (IQR) | 84 (79-89) | 80 (77-84) | 77 (75-82) |
| 75-79 | 19,417 (29.0) | 22,001 (45.9) | 31,543 (62.1) |
| 80-84 | 16,999 (25.4) | 15,079 (31.5) | 10,741 (21.1) |
| 85-89 | 16,442 (24.6) | 8161 (17.0) | 6011 (11.8) |
| 90+ | 14,001 (20.9) | 2675 (5.6) | 2526 (5.0) |
| Sex (female) | 40,029 (59.9) | 24,482 (51.1) | 24,461 (48.1) |
| Smoking status |  |  |  |
| Current smoker | 4897 (7.3) | 2861 (6.0) | 3386 (6.7) |
| Ex-smoker | 24,193 (36.2) | 21,872 (45.6) | 22,472 (44.2) |
| Non-smoker | 35,202 (52.7) | 22,925 (47.8) | 24,066 (47.4) |
| Missing | 2567 (3.8) | 258 (0.5) | 897 (1.8) |
| Alcohol intake |  |  |  |
| Current drinker | 35,117 (52.5) | 31,906 (66.6) | 31,196 (61.4) |
| Ex-drinker | 6559 (9.8) | 5570 (11.6) | 5282 (10.4) |
| Non-drinker | 10,971 (16.4) | 5711 (11.9) | 6883 (13.5) |
| Heavy or problem drinker | 1655 (2.5) | 1019 (2.1) | 1426 (2.8) |
| Missing | 12,557 (18.8) | 3710 (7.7) | 6034 (11.9) |
| Median weight in kg (IQR) | 69 (59-80) | 75 (65-86) | 76 (66-88) |
| Body mass index |  |  |  |
| <20 | 5278 (7.9) | 1799 (3.8) | 2141 (4.2) |
| 20 - <25 | 19,228 (28.8) | 12,494 (26.1) | 12,870 (25.3) |
| 25 - <30 | 18,282 (27.3) | 18,181 (37.9) | 17,370 (34.2) |
| 30 - <35 | 6865 (10.3) | 8347 (17.4) | 8196 (16.1) |
| ≥ 35 | 2564 (3.8) | 3372 (7.0) | 3542 (7.0) |
| Missing | 14,642 (21.9) | 3723 (7.8) | 6702 (13.2) |
| Mean CHA_2_DS_2_-VASc score (SD) | 4.2 (1.4) | 4.1 (1.3) | 4.5 (1.5) |
| Mean has-bled score (SD) | 3.3 (1.2) | 3.4 (1.3) | 3.0 (1.2) |
| Co-morbidities at study entry |  |  |  |
| Heart failure | 12,415 (18.6) | 5116 (10.7) | 13,307 (26.2) |
| Diabetes mellitus | 10,158 (15.2) | 7829 (16.3) | 10,053 (19.8) |
| Hypertension | 38,630 (57.8) | 31,845 (66.5) | 31,123 (61.2) |
| Ischaemic stroke, transient ischaemic attack, or thromboembolism | 13,084 (19.6) | 7805 (16.3) | 14,708 (28.9) |
| Coronary artery disease | 17,439 (26.1) | 12,248 (25.6) | 15,566 (30.6) |
| Peripheral vascular disease | 6349 (9.5) | 4194 (8.8) | 6568 (12.9) |
| Fragility fracture | 13,453 (20.1) | 7198 (15.0) | 7097 (14.0) |
| Heart valve replacement or mitral stenosis | 738 (1.1) | 626 (1.3) | 3072 (6.0) |
| Dementia | 6649 (9.9) | 909 (1.9) | 1998 (3.9) |
| Chronic renal impairment (any) | 13,141 (19.7) | 11,681 (24.4) | 10,784 (21.2) |
| Stage 1 | 41 (0.1) | 33 (0.1) | 49 (0.1) |
| Stage 2 | 499 (0.7) | 605 (1.3) | 587 (1.2) |
| Stage 3 | 9653 (14.4) | 9706 (20.3) | 8223 (16.2) |
| Stage 4 | 1023 (1.5) | 637 (1.3) | 698 (1.4) |
| End stage renal failure or dialysis | 291 (0.4) | 128 (0.3) | 154 (0.3) |
| Stage unknown | 1634 (2.4) | 572 (1.2) | 1073 (2.1) |
| Acute kidney injury | 1214 (1.8) | 2969 (6.2) | 2298 (4.5) |
| Liver disease | 980 (1.5) | 709 (1.5) | 804 (1.6) |
| Previous bleed (any) | 22,500 (33.7) | 16,857 (35.2) | 19,333 (38.0) |
| Major bleed | 2667 (4.0) | 1169 (2.4) | 1728 (3.4) |
| Clinically relevant non-major bleed | 20,825 (31.1) | 16,161 (33.7) | 18,349 (36.1) |
| Intracranial haemorrhage | 1340 (2.0) | 378 (0.8) | 649 (1.3) |
| Gastrointestinal bleed | 8172 (12.2) | 5939 (12.4) | 6086 (12.0) |
| Other bleed | 22,500 (33.7) | 16,857 (35.2) | 19,333 (38.0) |
| Median frailty score (IQR) | 0.22 (0.17-0.28) | 0.22 (0.17-0.28) | 0.22 (0.17-0.28) |
| Number of falls in past year |  |  |  |
| 0 | 60,311 (90.2) | 45,658 (95.3) | 48,139 (94.7) |
| 1-2 | 6133 (9.2) | 2198 (4.6) | 2574 (5.1) |
| ≥ 3 | 415 (0.6) | 60 (0.1) | 108 (0.2) |
| Mean number of GP encounters in year prior to study entry (SD) | 12.5 (10.9) | 11.7 (9.3) | 17.6 (13.5) |
| Medication prescribed within 3 months of study entry |  |  |  |
| Antiarrhythmics | 4777 (7.1) | 1881 (3.9) | 5944 (11.7) |
| Anticonvulsants | 2891 (4.3) | 1629 (3.4) | 2280 (4.5) |
| Antihypertensives | 49,418 (73.9) | 37,188 (77.6) | 44,112 (86.8) |
| Antiplatelets | 38,696 (57.9) | 24,782 (51.7) | 6654 (13.1) |
| Corticosteroids | 4883 (7.3) | 3674 (7.7) | 3159 (6.2) |
| Diabetic medications | 6301 (9.4) | 4963 (10.4) | 6701 (13.2) |
| Non-steroidal anti-inflammatories | 4282 (6.4) | 3258 (6.8) | 1259 (2.5) |
| Proton pump inhibitor or h_2_ receptor antagonist | 21,170 (31.7) | 15,447 (32.2) | 14,810 (29.1) |
| Selective serotonin reuptake inhibitors | 5752 (8.6) | 2329 (4.9) | 3465 (6.8) |
| Statins | 19,113 (28.6) | 20,627 (43.0) | 23,679 (46.6) |

*Results are presented as number (%) of patients, median (interquartile range), or mean (standard deviation). GP = general practice, CHA_2_DS_2_-VASc = stroke risk score, HAS-BLED = bleeding risk score.*

*Figure S1: Rate of prescribing of OACs in the AF population over time (/population count) with the red line indicating the mean prescribing as predicted by the Poisson regression model. The change points in the regression line show when DOACs were introduced to the UK market.*

**

*Figure S2. Risk difference of being prescribed an OAC for patients aged ≥ 75 years with a diagnosis of AF by smoking status*

*Figure S3. Risk difference of being prescribed an OAC for patients aged ≥ 75 years with a diagnosis of AF by alcohol status*

*Figure S4. Risk difference of being prescribed an OAC for patients aged ≥ 75 years with a diagnosis of AF by stroke and bleeding risk. Higher CHA2DS2-Vasc score = higher stroke risk; higher HAS-BLED score = higher bleeding risk.*

*Table S2. Univariate logistic regression model showing which characteristics and co-morbidities are associated with the prescribing of a DOAC over warfarin to patients aged ≥ 75 years with AF*

| Characteristic or co-morbidity | Hazard ratio | Lower 95% CI | Upper 95% CI | p-value |
| --- | --- | --- | --- | --- |
| Age 75-79 | 1 | 1 | 1 |  |
| Age 80-84 | 0.9948043 | 0.9289992 | 1.065271 | 0.881 |
| Age 85-89 | 1.229128 | 1.139558 | 1.325738 | <0.001 |
| Age 90+ | 2.015579 | 1.826251 | 2.224535 | <0.001 |
| BMI 20-24 | 1 | 1 | 1 | <0.001 |
| BMI < 20 | 1.185926 | 1.035332 | 1.358425 | 0.014 |
| BMI 25-29 | 0.8910896 | 0.83369 | 0.9524412 | 0.001 |
| BMI >30 | 0.850997 | 0.7880616 | 0.9189585 | <0.001 |
| Heart failure | 0.9316756 | 0.8627475 | 1.006111 | 0.071 |
| Hypertension | 0.9505645 | 0.8942575 | 1.010417 | 0.104 |
| Diabetes Mellitus | 0.9654399 | 0.9015461 | 1.033862 | 0.314 |
| Stroke/TIA/TE | 1.222453 | 1.143321 | 1.307063 | <0.001 |
| Coronary artery disease | 0.9174641 | 0.8611789 | 0.9774279 | 0.008 |
| Peripheral vascular disease | 1.008058 | 0.9156841 | 1.109752 | 0.87 |
| Chronic renal impairment | 1.032938 | 0.9743245 | 1.095078 | 0.277 |
| AKI | 1.404101 | 1.301472 | 1.514822 | <0.001 |
| Liver disease | 1.185166 | 0.963394 | 1.457989 | 0.108 |
| Any bleed | 1.062719 | 1.004443 | 1.124376 | 0.035 |
| Major bleed | 1.30063 | 1.103486 | 1.532995 | 0.002 |
| CRNMB bleed | 1.045219 | 0.9876041 | 1.106194 | 0.126 |
| Previous intracranial haemorrhage | 1.487242 | 1.142614 | 1.935815 | 0.003 |
| Previous gastrointestinal bleed | 1.040972 | 0.9646336 | 1.123351 | 0.301 |
| Previous other bleed | 1.062719 | 1.004443 | 1.124376 | 0.035 |
| Dementia | 2.631053 | 2.278766 | 3.037802 | <0.001 |
| Frailty score | 2.574146 | 1.952327 | 3.394015 | <0.001 |
| History of falls | 1.320283 | 1.241615 | 1.403936 | <0.001 |
| Fall in past year | 1.47352 | 1.310541 | 1.656766 | <0.001 |
| Fracture | 1.245837 | 1.160386 | 1.33758 | <0.001 |
| Heart valve disease | 0.5182116 | 0.4145209 | 0.6478402 | <0.001 |

*Table S3. Factors which remained significant in the multivariate model*

| Characteristic or co-morbidity | Hazard ratio | Lower 95% CI | Upper 95% CI | p-value |
| --- | --- | --- | --- | --- |
| Age 75-79 | 1 | 1 | 1 | 1 |
| Age 80-84 | 0.9554318 | 0.8898756 | 1.025818 | 0.209 |
| Age 85-89 | 1.112901 | 1.026683 | 1.206359 | 0.009 |
| Age 90+ | 1.818376 | 1.632569 | 2.02533 | 0 |
| Stroke/TIA/TE | 1.168816 | 1.086241 | 1.257668 | 0 |
| AKI | 1.41259 | 1.305653 | 1.528285 | 0 |
| Previous intracranial haemorrhage | 1.363572 | 1.0351 | 1.796278 | 0.027 |
| Dementia | 2.42026 | 2.07692 | 2.82036 | 0 |
| History of falls | 1.127135 | 1.046015 | 1.214545 | 0.002 |
| Fall in past year | 1.190888 | 1.043028 | 1.359709 | 0.01 |
| Fracture | 1.095025 | 1.013932 | 1.182603 | 0.021 |
| Heart valve disease | 0.5488154 | 0.4360888 | 0.6906814 | 0 |
